# Incorporation of Edutainment Into Intervention and Evaluation: The *Jump With Jill* (*JWJ*) Program

**DOI:** 10.3389/fpubh.2019.00163

**Published:** 2019-06-26

**Authors:** Jill Jayne

**Affiliations:** Jump with Jill, Pittsburgh, PA, United States

**Keywords:** edutainment, evaluation, nutrition education, elementary-aged children, social marketing

## Abstract

**Objective:** To incorporate media strategies (e.g., edutainment) into a pilot nutrition education intervention, *Jump with Jill* (*JWJ*), to (1) change student knowledge and intentions; (2) stimulate enthusiasm; and (3) enhance evaluation.

**Methods:** Third graders (*n* = 194) completed a pre-survey, attended a 60-min nutrition education show presented as a school assembly, and then completed a post-survey. Data was collected interactively, where students lined up behind an emoji that best represented their answers.

**Results:** Statistically significant improvements were reported for drink preferences and enjoyment of nutrition education as well as the experience of taking a survey. All aggregate responses for knowledge, attitudes and intention became significantly more positive (*p* = 0.05). Furthermore, ~95% reported positive ratings to participating in the survey (*P* < 0.0001).

**Discussion:** Use of edutainment may serve to stimulate change in nutrition knowledge and intention in 3rd grade students. Perfecting this teaching strategy into evaluations of health promotion programming may serve the field by increasing accuracy of student responses when language, reading level, or survey inexperience are barriers.

## Introduction

Through the use of entertaining strategies, mass media has been successful in impacting food intake in children (1, 2). Marketing techniques such as repetition, music, characters and excitement are well-thought-out, well-funded and incorporate theoretical frameworks from the fields of behavior change, education and social marketing (3). Use of entertainment as an educational tool, termed *edutainment* (4), has established guidelines (5). In brief, research-based messages are woven into the audiences' lived experiences (6) using compelling plots, songs, and characters. Storytelling, identification and attachment to strong characters, parasocial interaction, social modeling, competition and liking are incorporated to enhance attitudinal and behavioral change (3, 4). To be effective, edutainment messages should be sustained as part of a comprehensive campaign that connects learners to characters and stories showing behavior change while establishing a new social norm. Because health skills develop through practice, the positive experiences derived from learning through play help develop deep intrinsic motivations that positively influence health outcomes (7) and lead to high levels of participant program satisfaction (6). Edutainment has been a successful component of previous nutrition interventions, improving knowledge, attitude and intention (8–10) and developing resistance to persuasive messages (11).

Although amenable to change, habits and food preferences formed during childhood typically track into adulthood and may lead to development of chronic disease (12–14). Hence, it is important to utilize strategies directed at elementary-aged children that influence targeted food and beverage choices known to impact health, such as increasing intake of fruits, vegetables, and low-fat dairy foods as well as decreasing intake of sugar-sweetened beverages (15). However, successful nutrition education aimed at children cannot simply be didactic but should be interactive, enjoyable and developmentally appropriate (7, 16, 17). Moreover, programming should have a theoretical framework (7, 16, 17) such as those used in behavior change, education and social marketing (18). Finally, unique evaluation processes must be considered because children are mastering written language and may be inexperienced with surveys. Edutainment strategies integrated into evaluation protocol might be a tool to addresses this challenge.

Although schools are an obvious venue for educating children about healthy eating (16, 19, 20), many students receive inadequate nutrition education despite its success (21). While teachers consider nutrition education valuable for students (22), Common Core State Standards and high-stakes standardized testing make nutrition and physical education expendable (23). Limited resources (22) and lack of subject expertise make external nutrition education programs attractive (16). Employing media strategies can entice classroom teachers into including nutrition education, thereby overriding cost and time barriers (11).

Furthermore, reliable evaluation strategies are essential parts of any intervention. Assessment strategies for children are generally informal (using some type of written format) and focus on measuring changes in knowledge, attitude, and behavior. Such changes are especially difficult to assess in young children given their cognition levels (24). Moreover, with required standardized testing in elementary schools, questions have arisen regarding student stress in relation to any testing, (23, 25) generating interest in investigating alternative evaluation strategies.

**Figure 1:**
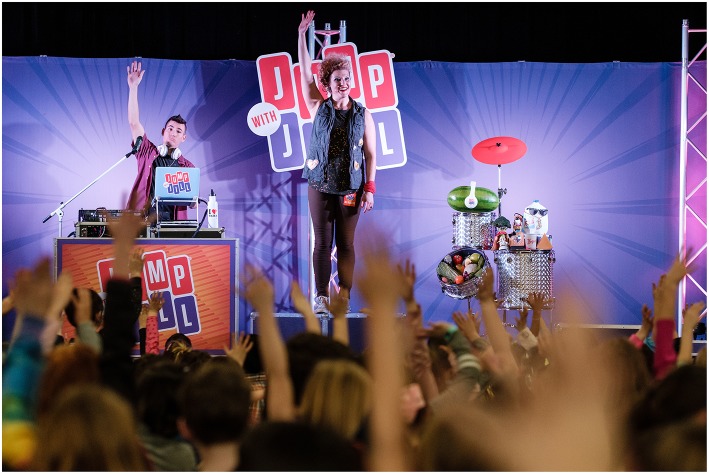
The music-based edutainment nutrition education program, JUMP WITH JILL (JWJ), engaging students and teachers in school-wide assembly.

Employing media strategies, *Jump with Jill* (*JWJ*) is a music-based, nutrition education program. *JWJ* creates a unique learning experience using original music, lighting, props, and live characters to inspire both children and teachers (Figure 1). During the 60-min school assembly, students dance and sing to behaviorally-focus songs that address increasing consumption of fruits, vegetables, dairy and water. Following the show, these characters and their messages can be used by classroom teachers via supplemental materials (e.g., music downloads, activity sheets, danceable music videos).

This study was undertaken to determine the impact of incorporating edutainment into both a nutrition education campaign and its evaluation. The hypotheses were such incorporation would (1) change student knowledge and intentions; (2) stimulate enthusiasm toward nutrition education; and (3) enhance the evaluation process.

## Methods

### Recruitment

An independent booking agency recruited schools for the 2015-2016 school year to receive *JWJ* at no cost. Eligible schools (*n* = 50) were within the 10 New Jersey counties that ranked lowest in the 2014 County Health Rankings (Atlantic, Camden, Cape May, Cumberland, Essex, Gloucester, Hudson, Passaic, Salem, and Union). One performance school per county was randomly chosen for evaluation. While the entire school attended the assembly, only one randomly-selected 3rd grade class completed surveys. Parental, student and teacher consent were handled at the district level. As all schools viewed *JWJ* as an adjunct to their education, the project was considered exempt from written consent. The University of Pittsburgh's Institutional Review Board approved this study as only deidentified evaluation data was used for the evaluation. No demographic information was collected.

### Survey Development

A survey was designed incorporating edutainment strategies to assess student knowledge, attitudes and intentions (e.g., questions repeated phrasing used in the show). For the first question, actual products (milk, water, energy drinks, fruit drinks, and soda) were displayed. Milk and Water were classified as a positive response (coded as 1) and Energy Drink/Fruit Drink/Soda (coded as 0) were classified as a negative response.

**Figure 2:**
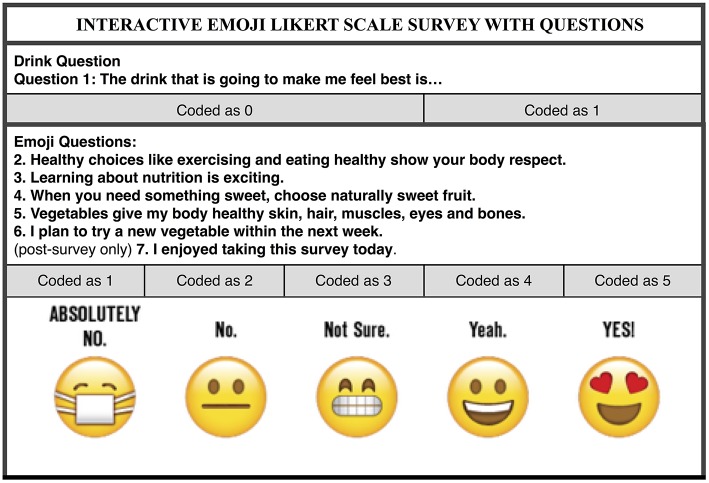
Integration of edutainment strategies into the evaluation of JUMP WITH JILL (JWJ), a nutrition education program.

For questions two through seven, instead of adapting more traditional “yummy/yucky faces” (26) or indication of “yes” or “no,” customized emojis were used to determine responses (Figure 2). To overcome obstacles associated with using a Likert Scale with children (27), emoji faces varied in relationship to the strength of response and were accompanied by words rather than numbers. In the *JWJ Likert Scale Survey Technique for Children*, responses were recorded such that each emoji represented a point on a 1 to 5 Likert scale with 1 reflecting “ABSOLUTELY NOT” and 5 reflecting “YES!.” In addition, at pre-test students were asked to raise their hands to determine previous experience with survey taking; at post-test, they were asked to stand in front of the emoji that reflected their survey enjoyment.

Prior to administration, cognitive testing with a small, convenience sample (*n* = 5; grades K-4) was conducted to determine how the expressions would be interpreted. Except for “No,” students indicated agreement with each face label. Originally a face with a tear depicted “No” but students thought that face represented “Sad.” The emoji was changed to a face with a line for a mouth.

### Survey Administration

**Figure 3:**
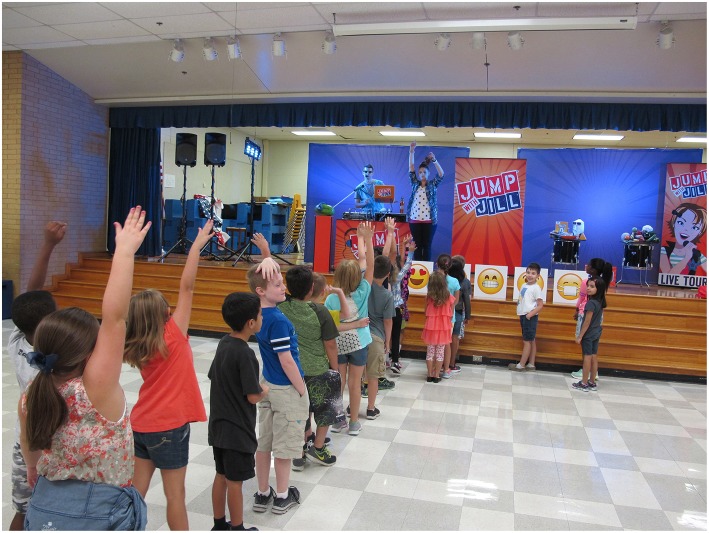
Third-grade students participating in the JUMP WITH JILL (JWJ) Likert Scale Survey Technique for Children. Written consent was obtained from the parents for all identifiable minors for the publication of this image.

Stations containing either a beverage (energy drink, fruit drink, soda, milk or water) (Question 1) or an emoji (Questions 2–7) were arranged. Before starting the survey, students were told by *JWJ* characters that there was no right answer and that the survey was not a test. To capitalize on the power of peer influence, students made a “Promise to be Honest” as a commitment to being thoughtful “research subjects” where their authentic opinions were valued. To minimize groupthink, students were asked to select their answers *before* moving to a station. Students then were asked to fully commit to their answer by lining up in front of the emoji that matched their response (Figure 3). The *JWJ* cast recorded the number of children at each station for each question. The students then joined the assembly. After the show, students reaffirmed their honesty pledge and repeated the pre-survey process.

### Data Analysis

Frequencies were calculated from recorded responses. Two types of outcomes were determined using aggregated data from all schools and time points (pre-test, post-test): the proportion of responses (children lining up behind an emoji) and an overall score. The proportion of responses was calculated as: sum of children lining up behind an emoji divided by total of children multiplied by 100. Pairwise differences among dependent proportions were tested using simultaneous 95% confidence intervals among dependent proportions (assuming a multinomial distribution) (28, 29). The overall score was calculated (for all but question 1) by multiplying the emoji's response choice (1–5) by the number of children lining up behind it, then summing all multiples for each time point and school. Higher overall scores indicated more positive responses. Differences in scores between pre- and post-tests were assessed. Distribution of overall scores and differences were determined to be non-normal; hence, the Wilcoxon Signed-Ranks test was conducted using Statistical Analysis Software (version 9.3, 2011, SAS Institute Inc, Cary, NC).

## Results

*JWJ* performed 50 shows reaching nearly 14,700 elementary school children. Over 75% of the 194 3rd graders participating in the evaluation reported no previous experience with taking a prior survey. Following the show, statistically significant differences in individual scores were noted for questions 1, 3, 4, and 5 (Table 1). Moreover, all aggregate responses became significantly more positive (Table 2). Furthermore, ~85% reported the highest positive rating (YES!) to survey participation, and 95% reported positive ratings (YES! or Yeah), which differed significantly from those reporting negative ratings (ABSOLUTELY NOT and No) (*P* < 0.0001).

**Table 1:** Proportions of individual score values from third grade students (*n* = 194) at 10 NJ schools after attending the *Jump with Jill* show (School Year 2015-16)^a^.

**Question**	**Response choice**	**Pre-test**		**Post-test**	
		***n***	**%**	***n***	**%**
1. The drink that is going to make me feel best is…	Milk, water	130	66.3	168	87.1^*^
	Soda, fruit drink, energy drink	66	33.7	25	12.9
2. Healthy choices like exercising and eating healthy show your body respect.	Absolutely no	9	4.6	5	2.6
	No	2	1.0	0	0
	Not sure	17	8.8	10	5.2
	Yeah	50	25.8	39	20.1
	Yes	116	59.8	140	72.2
3. Learning about nutrition is exciting.	Absolutely no	63	32.8	18	9.3^**^
	No	13	6.8	5	2.6
	Not sure	22	11.5	40	20.6
	Yeah	39	20.3	33	17.0
	Yes	55	28.7	98	50.5^*^
4. When you need something sweet, choose naturally sweet fruit.	Absolutely no	34	17.5	13	6.7
	No	16	8.2	17	8.8^**^
	Not sure	43	22.2	34	17.5
	Yeah	26	13.4	28	14.4
	Yes	75	38.7	102	52.6
5. Vegetables give my body healthy skin, hair, muscles, eyes, and bones.	Absolutely no	18	9.3	0	0^**^
	No	6	3.1	2	1.0
	Not sure	31	15.9	22	11.3
	Yeah	45	23.2	30	15.5
	Yes	94	48.5	140	72.2
6. I plan to try a new vegetable within the next week.	Absolutely no	9	4.6	5	2.6
	No	2	1.0	0	0
	Not sure	17	8.8	10	5.2
	Yeah	50	25.8	39	20.1
	Yes	116	59.8	140	72.2

a *Scores were determined by having children line up behind an emoji depicting the following responses equated with the corresponding value: Absolutely no = 1; No = 2; Not Sure 3; Yeah = 4; Yes = 5. Proportions were determined by dividing the number of children who lined up behind the emoji representing that response choice by the total number of children who lined up behind all emojis for that questions and time point. For Question 1 (drinks), children that lined up behind Milk and Water were combined and children who lined up behind the Energy Drink, Fruit Drink, and Soda were combined prior to calculating the proportions. P-values were determined by obtaining simultaneous 95% confidence intervals for pairwise differences among the dependent proportions assuming a multinomial distribution*.

* p < 0.05;

** *p < 0.01*.

**Table 2:** Mean of overall scores from third grade students (*n* = 194) at 10 NJ schools before and after attending the *Jump with Jill* show (School Year 2015-16)^a^.

**Question**	**Pre-survey (x ± SD)**	**Post-survey (x ± SD)**	**S (signed Rank)**	***P*-value**
1. The drink that is going to make me feel best is…				
Milk, water	13.0 ± 4.83	16.8 ± 4.76	25.5	0.008^**^
Soda, Fruit Drink, Energy Drink	6.6 ± 3.89	2.5 ± 2.84	−26	0.006^**^
2, Healthy choices like exercising and eating healthy show your body respect.	84.4 ± 18.61	89.1 ± 15.9	18	0.008^**^
3. Learning about nutrition is exciting.	58.6 ± 20.82	77.0 ± 13.22	22.5	0.019^*^
4. When you need something sweet, choose naturally sweet fruit.	67.4 ± 21.38	77.1 ± 16.52	20.5	0.016^*^
5. Vegetables give my body healthy skin, hair, muscles, eyes and bones.	77.3 ± 15.97	89.0 ± 13.19	27.5	0.002^**^
6. I plan to try a new vegetable within the next week.	56.3 ± 10.87	64.7 ± 12.28	22.5	0.004^**^
7. I enjoyed taking this survey today.		92.8 ± 15.22		

a *Scores were determined by having children line up behind an emoji depicting the following responses equated with the corresponding value: Absolutely no = 1; No = 2; Not Sure 3; Yeah = 4; Yes = 5. Score value was calculated at each time point by first multiplying each question's score by the number of children who lined up behind that question, then summing these multiples for each time point and school. For the question 1, scores for Milk and Water were combined and classified as a positive response; Energy Drink/Fruit Drink/Soda were combined and classified a negative response. P-values were determined using the Wilcoxon Signed-Ranks test*.

* p < 0.05;

** *p < 0.01*.

## Discussion

Overall, using media strategies associated with edutainment to change student knowledge and intention as well as to detect these changes was confirmed. Use of enthusiastic endorsement of the expected behaviors (a Social Learning Theory component) by *JWJ* characters (a marketing strategy) appeared to stimulate change in nutrition knowledge and intention in these 3rd grade students. These findings confirm previous observations that theatrical interventions in schools can be successful (8–10, 30, 31).

*JWJ* creates positive, realistic impressions of healthy foods, moderates negative responses, and creates commitment for those that start out as “not sure.” *Jump with Jill* deconstructs the framework that kids have built with their dislikes and breaks ground with newfound aspirational attitudes, as seen by the decrease in the number of extreme negative responses for all questions. The positive experience derived from *JWJ* helps audiences develop deep intrinsic motivations that lead to high levels of program satisfaction among participants. Moreover, through the many touch points of the program, the students and their teachers attach to the characters. Their relationship deepens as they go from interacting with the show to viewing videos to working in small groups with the characters to complete the survey where their opinion is valued and documented.

While the positive results of the intervention are exciting, a major focus of this pilot study was developing an evaluation tool that aligned with the educational approach of the program. Unique to this study was the integration of media strategies into the evaluation process. Edutainment techniques successfully captured changes in 3rd grade students' knowledge and intention. Making the evaluation process interactive and fun (game-like) resulted in over 95% of students enjoying participation in a study, in contrast to reports of student stress in relation to testing (32, 33). A positive experience can have meaningful measurements for likelihood of healthy behaviors. Researchers have noted high intensity emotional experiences has an increased influence on behavior, so strong it can even overwhelm cognitive processing (34). Nutrition education in the form of a rock show is a strategic choice to empower audiences to action. Additionally, children in 3rd grade are in Piaget's concrete operational phase of cognitive development, where they begin to use inductive logic and become less egocentric (35).

Using the “Promise to be Honest” pledge built upon their developing senses of having unique thoughts of fairness (35) and decreased social desirability. Students were proud to be forthright with their opinions even though they were aired in front of their peers. Setting clear expectations for behavior (aka defining the rules of the game) is crucial. It is suggested that only a small, familiar group (e.g., one class) be used for this data collection method as the researchers experienced a diminished effect of accountability as group sizes grew. For example, as this methodology is scaled to other *JWJ* projects and sample size is increased, we are still evaluating classrooms one at a time rather than combining classes. Adjustments in the show and travel schedule are made to allow for these multiple classroom visits before and after the show as part of the intervention design.

Perfecting edutainment within nutrition education evaluations in elementary schools may serve the field by increasing accuracy of student responses when language, reading level, or survey inexperience are barriers. These study participants saw the evaluation as part of the intervention, hence creating a positive experience throughout appears to enhance satisfaction. Furthermore, this was an intentionally simple evaluation study design that could fit into the parameters of a school day, requiring no additional staffing from the school or *JWJ*.

Several limitations must be acknowledged. This current study lacks follow-up to demonstrate students' knowledge retention or behavior change. Other limitations include lack of a control group and unpaired pre- and post- survey data, both of which restrict generalizability. Furthermore, statistically significant proportions that change by a fraction of a decimal to a few points must be interpreted with caution.

## Conclusions and Implications

The mass media has proven successful at impacting food intake in children (1, 2). If “celebrities” can shape social norms about unhealthy behaviors, then nutrition educators might be able to use these same strategies to shift youth toward healthy behaviors. If children demand high quality entertainment, then health-related media can compete. The media could be the best guide in developing solutions to address youth health. Suggestions for best practices in incorporating this novel evaluation strategy into other programs include: working in small groups, for example, by classroom; pausing to let students decide answers before reporting to the group; decreasing test anxiety and reducing test fatigue by making the evaluation into a game; and decreasing social desirability bias by using a “promise to be honest” pledge.

To provide additional validation for using media strategies in nutrition education, along with the impact of *JWJ* on food choice behavior, future studies including a comparison group, more follow-up data points, demographic characteristics of children, and other confounding variables are warranted.

## Data Availability

The datasets generated for this study are available on request to the corresponding author.

## Ethics Statement

This study was carried out in accordance with the recommendations of the school districts involved in the study. Parental, student and teacher consent were handled at the district level. As all schools viewed *JWJ* as an adjunct to their education, the project was considered exempt from written consent. The University of Pittsburgh's Institutional Review Board approved this analytical portion of the study as only deidentified evaluation data was used. No demographic information was collected. The University of Pittsburgh Institutional Review Board approved this study.

## Author Contributions

The author confirms being the sole contributor of this work and has approved it for publication.

### Conflict of Interest Statement

JJ holds all trademarks, copyrights, and likenesses associated with the property, Jump with Jill.
